# 

**Published:** 1909-10-02

**Authors:** 


### GIFTS AND BEQUESTS.

Miss Emma Smith, of Cavendish Road, Leeds, by her will left £100 to the Leeds Workpeople's Hospital Fund.

The Treasurers of the Middlesex Hospital have received from Miss B. Wotton a gift of £500 Consols for the benefit of the Cancer Charity of the Hospital.

The Treasurer of St. Bartholomew's Hospital has received £2,500, being moiety of the legacy left by Mrs. A. J. Holborn to the funds of the hospital.

Miss Harriet Davie Lancaster, of Leamington, who died on August 15, bequeathed £100 each to the Kenilworth Convalescent Hospital, the Warneford Hospital, and the Midland Counties Home for Incurables.

Montrose Infirmary has received a gift of £300 from the widow of General Henry Renny, C.S.I., 81st Regiment. The money is to be devoted to the dedication of a bed in the institution in memory of Alex. Tailyour Renny and Henry Thomas Renny, sons of the late General and Mrs. Renny.

The late Mr. John Hogg, of Paternoster Row, left, subject to his wife's interest, £500 each to Sir William Treloar's Home for Cripples and the Home for the Dying (Clapham). The ultimate residue of his estate the testator left upon trust; but in the event of the failure of this he bequeathed £600 to the Scottish Hospital in London; £250 to the Surgical Aid Society; and £500 to the Royal Boscombe and West Hants Hospital.

### The Question Box.

It has been represented to us that it would fulfil a useful purpose to devote space to a Question Box Department where any matter of interest to hospital workers could be raised when a difficulty arose in a particular institution. In this way it is felt that the experience of the best-managed hospitals can be made helpful to the whole hospital world. We hope our readers may approve this new departure, and use the Question Box whenever an opportunity offers. We invite the contribution of both questions and answers.

We submit a few questions which have been asked, and shall be glad to receive answers to them in due course. Answers if published will be paid for at Scale rates.

*The Honorary Medical Staff.*

1. Is it possible to limit the visits of attending physicians and surgeons to certain specified hours?

*Nursing.*

2. Is the eight hours' day for nurses practicable?

*Accounts.*

3. Should ordinary repairs be treated in the accounts under the head of Ordinary or Extraordinary Expenditure?

*Finance.*

4. Has the time arrived when each voluntary hospital where the ticket system prevails should make such tickets represent a subscription at least equal to the cost to the hospital of the treatment given to the in- and out-patient to whom this may be given?

*Ambulance.*

5. Is there a satisfactory automobile ambulance yet on the market?

*Hospitals and the Press.*

6. What privileges should be accorded to the Press in regard to hospital cases, and especially to accident cases?

*Hospital Administration.*

7. Is it desirable that a chair of Hospital Administration be established at one or more of the Universities?

*Poor Law Infirmaries.*

8. Should Poor Law Infirmaries have a Visiting Medical Staff? If so, on what terms and conditions of attendance?

### APPOINTMENTS VACANT.

Surrey Dispensary, Southwark, S.E.—Physician.
London Hospital, Whitechapel, E.—Assistant Surgeon.
London Hospital, Whitechapel, E.—Surgeon.
Farringdon General Dispensary and Lying-in Charity, Holborn Circus.—Resident Medical Officer.

### THE GRADUATES' MEDICAL SCHOOLS.

DIARY FOR THE WEEK, OCTOBER 4 to 9.

### MEDICAL GRADUATES' COLLEGE AND POLYCLINIC, 22 Chenies Street, W.C.

**Clinique.**

**At 4 p.m.**

**October 4**, Dr. Colcott Fox, **Skin.**
**October 5**, Dr. J. E. Squire, **Medicine.**
**October 6**, Mr. Mower White, **Surgery.**
**October 7**, Sir Jonathan Hutchinson, **Surgery.**
**October 8**, Mr. Sydney Stephenson, **Eye.**

**At 5.15 p.m.**

**October 4**, Prof. J. H. Nicoll (Glasgow), **The Treatment of Senile Enlargement of the Prostate.**

**October 5**, Dr. James Donelan, **Suppuration in the Accessory Cavities of the Nose.**

**October 6**, Mr. MacLeod Yearsley, **The Nose and Ear in School Medical Inspection.**

**October 7**, Mr. Jackson Clarke, **A Series of Abdominal Cases.**

### THE POST-GRADUATE COLLEGE, West London Hospital, Hammersmith, W.

**At 10 a.m.**

**October 4 and 7**, Surgical Registrar, **Demonstration of Cases.**

**At 12 noon.**

**October 7**, Dr. Bernstein, **Pathological Demonstration.**

**At 12.15 p.m.**

**October 5**, Dr. Pritchard, **Practical Medicine.**
**October 6**, Dr. Grainger Stewart, **Practical Medicine.**

**At 5 p.m.**

**October 5.** Dr. Saunders, **Clinical Examination of Severe Cases of Gastric Disorder.**
**October 6**, Dr. Beddard, **Medicine.—I.**
**October 8**, Mr. Etherington-Smith, **Clinical Lecture.**

The following appointments at the Edinburgh Royal Infirmary are announced : *Resident Physicians for six months:* —Alexander Gibson, M.A., M.B., Ch.B.; G. E. Mackay, M.B., Ch.B.; James Langwill, M.B., Ch.B.; John Ware, M.B., Ch.B.; H. Ruthven Lawrence, M.B., Ch.B.; J. M. Deuchars, M.B., Ch.B. *Resident Surgeons.*—Frank E. Jardine, M.B., Ch.B.; R. Charles Alexander, M.A., M.B., Ch.B.; Spencer Jackson, M.B., Ch.B.; Fred R. Laing, M.B., Ch.B.; Adam White, M.B., Ch.B.; Robert C. Harkness, M.B., Ch.B. *Non-Resident House Surgeon.*—A. W. Burton, M.B., Ch.B. *Clinical Assistants.*—J. J. M. Shaw, M.B., Ch.B.; Kenneth Fraser, M.B., Ch.B.; J. K. M. Dickie, M.B., Ch.B.; Leonard Leslie, M.B., Ch.B.; H. Faithful Smith, M.B., Ch.B.; William F. Christie, M.B., Ch.B.; R. W. Lang Todd, M.B., Ch.B., B.Sc.; Norman S. Carmichael, M.B., Ch.B. (medical out-patient department); F. W. Hay, M.B., Ch.B.; J. M. M'Keand, M.B., Ch.B.; L. E. Barrington-Wood, M.B., Ch.B.; W. M. Menzies, M.B., Ch.B.; A. Waugh Young, M.B., Ch.B., F.R.C.S.Ed.; Miss Marguerite Ross, M.B., Ch.B.; Miss Lydia K. Towers, M.B., Ch.B.

THE HOSPITAL October 2, 1909.

***Name*** ..........................................................

***Address*** ..........................................................

..........................................................

This Coupon must accompany manuscript or contributions intended for The Hospital.

